# 3,4,5-Trihy­droxy­benzoic acid

**DOI:** 10.1107/S1600536811007471

**Published:** 2011-03-05

**Authors:** Namon Hirun, Saowanit Saithong, Chaveng Pakawatchai, Vimon Tantishaiyakul

**Affiliations:** a Department of Pharmaceutical Chemistry, Faculty of Pharmaceutical Sciences, Prince of Songkla University, Hat Yai, Songkhla 90112, Thailand; b Department of Chemistry, Faculty of Science, Prince of Songkla University, Hat Yai, Songkhla 90112, Thailand

## Abstract

In the title compound, C_7_H_6_O_5_, the three hy­droxy groups on the ring are oriented in the same direction. There are two intra­molecular O—H⋯O hydrogen bonds in the ring. In the crystal, there are several inter­molecular O—H⋯O hydrogen bonds and a short contact of 2.7150 (18) Å between the O atoms of the *para*-OH groups of adjacent mol­ecules.

## Related literature

For the biological activity of the title compound, see: Gomes *et al.* (2003 ▶); Priscilla & Prince (2009 ▶); Lu *et al.* (2010 ▶). For the structure of gallic acid monohydrate, see: Okabe *et al.* (2001 ▶); Jiang *et al.* (2000 ▶); Billes *et al.* (2007 ▶).
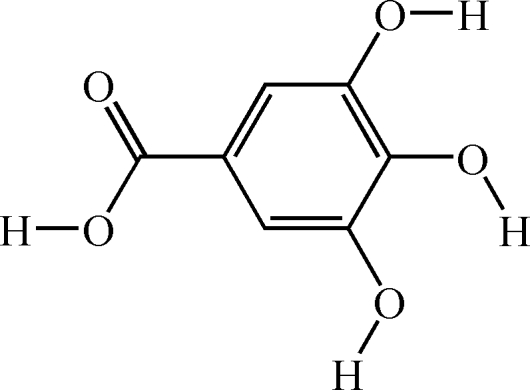


## Experimental

### 

#### Crystal data

C_7_H_6_O_5_
*M* *_r_* = 170.12
Monoclinic, 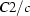

*a* = 25.629 (2) Å
*b* = 4.9211 (4) Å
*c* = 11.2217 (9) Å
β = 106.251 (1)°
*V* = 1358.77 (19) Å^3^
*Z* = 8
Mo *K*α radiation
μ = 0.15 mm^−1^
*T* = 293 K
0.30 × 0.19 × 0.11 mm

#### Data collection

Bruker APEX CCD area-detector diffractometer
Absorption correction: multi-scan (*SADABS*; Bruker, 2003 ▶) *T*_min_ = 0.916, *T*_max_ = 1.000
7171 measured reflections
1254 independent reflections
1172 reflections with *I* > 2s(*I*)
*R*_int_ = 0.022

#### Refinement

*R*[*F* ^2^ > 2σ(*F* ^2^)] = 0.032
*wR*(*F* ^2^) = 0.091
*S* = 1.06
1254 reflections
121 parameters
4 restraints
H atoms treated by a mixture of independent and constrained refinement
Δρ_max_ = 0.16 e Å^−3^
Δρ_min_ = −0.21 e Å^−3^

### 

Data collection: *SMART* (Bruker, 1998 ▶); cell refinement: *SAINT* (Bruker, 2003 ▶); data reduction: *SAINT*; program(s) used to solve structure: *SHELXS97* (Sheldrick, 2008 ▶); program(s) used to refine structure: *SHELXL97* (Sheldrick, 2008 ▶); molecular graphics: *SHELXTL* (Sheldrick, 2008 ▶) and *Mercury* (Macrae *et al.*, 2008) ▶; software used to prepare material for publication: *SHELXL97* and *publCIF* (Westrip, 2010 ▶).

## Supplementary Material

Crystal structure: contains datablocks I, global. DOI: 10.1107/S1600536811007471/fj2397sup1.cif

Structure factors: contains datablocks I. DOI: 10.1107/S1600536811007471/fj2397Isup2.hkl

Additional supplementary materials:  crystallographic information; 3D view; checkCIF report

## Figures and Tables

**Table 1:** Hydrogen-bond geometry (Å, °)

*D*—H⋯*A*	*D*—H	H⋯*A*	*D*⋯*A*	*D*—H⋯*A*
O2—H2⋯O1	0.82 (1)	2.19 (2)	2.6625 (14)	117 (1)
O3—H3⋯O2	0.82 (1)	2.35 (2)	2.7464 (14)	110 (1)
O1—H1⋯O5^i^	0.84 (1)	1.89 (2)	2.7324 (13)	176 (2)
O3—H3⋯O3^ii^	0.82 (1)	2.04 (2)	2.8167 (9)	157 (2)
O4—H4⋯O5^iii^	0.85 (2)	1.81 (2)	2.6570 (13)	175 (2)

Symmetry codes: (i) 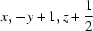; (ii) 
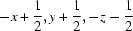
; (iii) 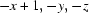.

**Fig. 1.**
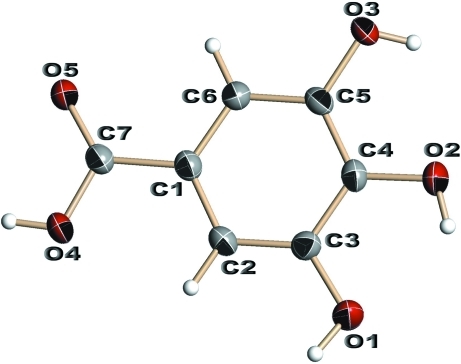
Molecular structure of I with thermal ellipsoids plotted at the 30% probability level.

**Fig. 2.**
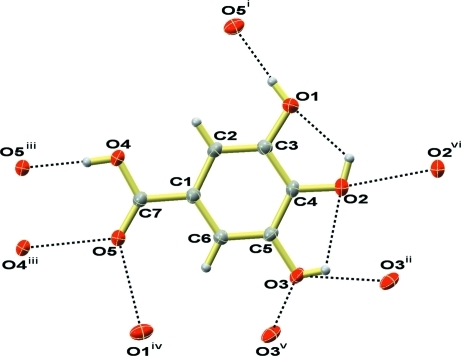
The intra- and inter hydrogen bonds of I are shown. Symmetry code: i = x, 1 - y, 1/2 + z; ii = 1/2 - x, 1/2 + y, z - 1/2; iii = 1 - x, -y, -z; iv = x, 1 - y, z - 1/2; v = 1/2 - x, y - 1/2, -z - 1/2; vi = 1/2 - x, 2.5 - y, -z.

**Fig. 3.**
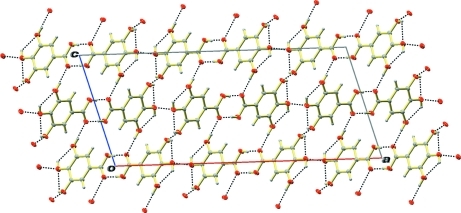
The packing interactions plotted down the b axis.

## supplementary crystallographic information

### Comment

Gallic acid, 3,4,5-trihydroxybenzoic acid, has been reported to have various biological activities such as antioxidant, antimutagenic, anticarcinogenic, antihyperglycemic and cardioprotective effects (Gomes *et al.*, 2003; Priscilla & Prince, 2009; Lu *et al.*, 2010). It has been shown that the activity of polyphenolic compounds, including gallic acid, is dependent on their structural characteristics (Gomes *et al.*, 2003). Thus, the investigation of its crystal structure is important for a better understanding of its biological functions. Recently, different crystal structures of gallic acid monohydrate have been reported (Jiang *et al.*, 2000; Okabe *et al.*, 2001; Billes *et al.*, 2007). Here, for the first time, the crystal structure of anhydrous gallic acid (I) was determined. The molecular structure of I is planar [Fig.1]. All the H atoms of the three hydroxy groups are oriented in the same direction.

The intra-hydrogen bonds are found between these hydroxy groups, O2···O1 = 2.6625 (14) and O3···O2 = 2.7464 (14)Å [Table.1]. This agrees with the report of Okabe *et al.* (2001). However, this orientation is inconsistent with those described by Billes *et al.* (2007) and Jiang *et al.* (2000), in which one H atom of the hydroxy group is oriented in the opposite direction to the others. The dissimilarity between I and the gallic acid monohydrate structure reported by Okabe is the different orientation of their carboxyl groups in relation to the direction of the three hydroxy groups.

The inter-hydrogen bonds in the crystal packing of I are found between oxygen atoms,O3···O3^ii^ [2.8167 (9) Å, symmetry code (ii): 1/2 - *x*, *y* + 1/2 - *z* - 1/2], O1···O5^i^ [2.7324 (13) Å, symmetry code (i): *x*, -*y* + 1, *z* + 1/2] and O4···O5^iii^ [2.6570 (13) Å, symmetry code (iii): -*x* + 1, -*y*, -*z*] [Table 1]. Moreover, the short contact between the oxygen of the hydroxy groups of the adjacent molecule is observed, O2···O2^vi^ [2.7150 (18) Å, symmetry code (vi): 1/2 - *x*, 2.5 - *y*, -*z*]. All intra- and intermolecular interactions including short contacts are depicted in Fig. 2 and the packing interactions as plotted down the *b* axis are shown in Fig. 3.

### Experimental

Gallic acid monohydrate was obtained from Fluka Chemie GmbH (Buchs, Switzerland). The anhydrous gallic acid crystals for this X-ray structure study were obtained by dissolving gallic acid monohydrate in diethyl ether followed by a slow evaporation of the solvent.

### Refinement

The structure was solved by direct methods refined by a full-matrix least-squares procedure based on *F^2^*. All hydrogen atoms of oxygen atoms were located in a difference Fourier map and restrained to ride on their parent atoms, O—H = 0.82–0.85 Å with *U*_iso_(H) = 1.2*U*_eq_(O). The hydrogen atoms of C-*sp*^2^ atom are constrained, C—H = 0.96 Å with *U*_iso_(H) = 1.2*U*_eq_(C), respectively.

### Crystal data

**Table:**

C_7_H_6_O_5_	*F*(000) = 704
*M**_r_* = 170.12	*D*_x_ = 1.663 Mg m^−^^3^
Monoclinic, *C*2/*c*	Mo *K*α radiation, λ = 0.71073 Å
Hall symbol: -C 2yc	Cell parameters from 3476 reflections
*a* = 25.629 (2) Å	θ = 3.3–28.1°
*b* = 4.9211 (4) Å	µ = 0.14 mm^−^^1^
*c* = 11.2217 (9) Å	*T* = 293 K
β = 106.251 (1)°	Hexagon, colourless
*V* = 1358.77 (19) Å^3^	0.30 × 0.19 × 0.11 mm
*Z* = 8	

### Data collection

**Table:**

Bruker APEX CCD area-detector diffractometer	1254 independent reflections
Radiation source: fine-focus sealed tube	1172 reflections with *I* > 2s(*I*)
graphite	*R*_int_ = 0.022
Frames, each covering 0.3 ° in ω scans	θ_max_ = 25.5°, θ_min_ = 1.7°
Absorption correction: multi-scan (*SADABS*; Bruker, 2003)	*h* = −30→30
*T*_min_ = 0.916, *T*_max_ = 1.000	*k* = −5→5
7171 measured reflections	*l* = −13→13

### Refinement

**Table:**

Refinement on *F*^2^	Primary atom site location: structure-invariant direct methods
Least-squares matrix: full	Secondary atom site location: difference Fourier map
*R*[*F*^2^ > 2σ(*F*^2^)] = 0.032	Hydrogen site location: inferred from neighbouring sites
*wR*(*F*^2^) = 0.091	H atoms treated by a mixture of independent and constrained refinement
*S* = 1.06	*w* = 1/[σ^2^(*F*_o_^2^) + (0.054*P*)^2^ + 0.7476*P*] where *P* = (*F*_o_^2^ + 2*F*_c_^2^)/3
1254 reflections	(Δ/σ)_max_ < 0.001
121 parameters	Δρ_max_ = 0.16 e Å^−^^3^
4 restraints	Δρ_min_ = −0.21 e Å^−^^3^

### Special details

**Table:**

Geometry. All e.s.d.'s (except the e.s.d. in the dihedral angle between two l.s. planes) are estimated using the full covariance matrix. The cell e.s.d.'s are taken into account individually in the estimation of e.s.d.'s in distances, angles and torsion angles; correlations between e.s.d.'s in cell parameters are only used when they are defined by crystal symmetry. An approximate (isotropic) treatment of cell e.s.d.'s is used for estimating e.s.d.'s involving l.s. planes.
Refinement. Refinement of *F*^2^ against all reflections. The weighted *R*-factor *wR* and goodness of fit *S* are based on *F*^2^, conventional *R*-factors *R* are based on *F*, with *F* set to zero for negative *F*^2^. The threshold expression of *F*^2^ > 2*σ*(*F*^2^) is used only for calculating *R*-factors(gt) *etc*. and is not relevant to the choice of reflections for refinement. *R*-factors based on *F*^2^ are statistically about twice as large as those based on *F*, and *R*- factors based on all data will be even larger.

### Fractional atomic coordinates and isotropic or equivalent isotropic displacement parameters (Å^2^)

**Table:**

	*x*	*y*	*z*	*U*_iso_*/*U*_eq_	
C1	0.40390 (5)	0.4849 (2)	−0.00701 (11)	0.0251 (3)	
C2	0.40861 (5)	0.6475 (3)	0.09733 (11)	0.0267 (3)	
H2A	0.4380	0.6271	0.1674	0.032*	
C3	0.36900 (5)	0.8389 (3)	0.09502 (11)	0.0256 (3)	
C4	0.32438 (5)	0.8667 (2)	−0.00919 (11)	0.0249 (3)	
C5	0.32041 (5)	0.7058 (2)	−0.11289 (11)	0.0247 (3)	
C6	0.36006 (5)	0.5156 (3)	−0.11223 (12)	0.0262 (3)	
H6A	0.3575	0.4083	−0.1819	0.031*	
C7	0.44558 (5)	0.2786 (2)	−0.00657 (11)	0.0262 (3)	
O1	0.36873 (4)	1.0116 (2)	0.18952 (9)	0.0367 (3)	
H1	0.3935 (6)	0.969 (4)	0.2539 (15)	0.044*	
O2	0.28434 (4)	1.0485 (2)	−0.01206 (10)	0.0352 (3)	
H2	0.2920 (6)	1.127 (3)	0.0553 (13)	0.042*	
O3	0.27680 (4)	0.7268 (2)	−0.21619 (9)	0.0334 (3)	
H3	0.2610 (7)	0.872 (3)	−0.2151 (16)	0.040*	
O4	0.48474 (4)	0.2647 (2)	0.09722 (9)	0.0392 (3)	
H4	0.5076 (7)	0.143 (3)	0.0928 (17)	0.047*	
O5	0.44465 (4)	0.12950 (19)	−0.09537 (8)	0.0314 (3)	

### Atomic displacement parameters (Å^2^)

**Table:**

	*U*^11^	*U*^22^	*U*^33^	*U*^12^	*U*^13^	*U*^23^
C1	0.0237 (6)	0.0240 (6)	0.0263 (6)	0.0027 (5)	0.0050 (5)	0.0020 (5)
C2	0.0244 (6)	0.0292 (7)	0.0234 (6)	0.0041 (5)	0.0015 (5)	0.0025 (5)
C3	0.0284 (6)	0.0248 (6)	0.0230 (6)	0.0007 (5)	0.0062 (5)	0.0000 (5)
C4	0.0224 (6)	0.0219 (6)	0.0301 (7)	0.0036 (5)	0.0070 (5)	0.0028 (5)
C5	0.0214 (6)	0.0236 (6)	0.0254 (6)	−0.0008 (5)	0.0005 (5)	0.0018 (5)
C6	0.0260 (6)	0.0252 (6)	0.0256 (6)	0.0021 (5)	0.0042 (5)	−0.0027 (5)
C7	0.0238 (6)	0.0264 (7)	0.0263 (6)	0.0028 (5)	0.0037 (5)	0.0014 (5)
O1	0.0420 (6)	0.0387 (6)	0.0251 (5)	0.0130 (4)	0.0020 (4)	−0.0060 (4)
O2	0.0309 (5)	0.0345 (6)	0.0369 (5)	0.0131 (4)	0.0038 (4)	−0.0051 (4)
O3	0.0265 (5)	0.0293 (5)	0.0346 (5)	0.0063 (4)	−0.0074 (4)	−0.0050 (4)
O4	0.0332 (5)	0.0433 (6)	0.0324 (5)	0.0189 (4)	−0.0054 (4)	−0.0078 (4)
O5	0.0292 (5)	0.0322 (5)	0.0289 (5)	0.0101 (4)	0.0019 (4)	−0.0037 (4)

### Geometric parameters (Å, °)

**Table:**

C1—C6	1.3918 (17)	C5—O3	1.3706 (14)
C1—C2	1.3951 (18)	C5—C6	1.3800 (18)
C1—C7	1.4726 (17)	C6—H6A	0.9300
C2—C3	1.3796 (18)	C7—O5	1.2325 (15)
C2—H2A	0.9300	C7—O4	1.3093 (15)
C3—O1	1.3606 (15)	O1—H1	0.844 (14)
C3—C4	1.3949 (17)	O2—H2	0.821 (14)
C4—O2	1.3551 (15)	O3—H3	0.824 (14)
C4—C5	1.3874 (18)	O4—H4	0.850 (15)
			
C6—C1—C2	120.64 (11)	O3—C5—C4	121.15 (11)
C6—C1—C7	119.36 (11)	C6—C5—C4	120.10 (11)
C2—C1—C7	120.00 (11)	C5—C6—C1	119.77 (12)
C3—C2—C1	119.02 (11)	C5—C6—H6A	120.1
C3—C2—H2A	120.5	C1—C6—H6A	120.1
C1—C2—H2A	120.5	O5—C7—O4	121.64 (11)
O1—C3—C2	125.17 (11)	O5—C7—C1	123.91 (11)
O1—C3—C4	114.22 (11)	O4—C7—C1	114.45 (11)
C2—C3—C4	120.60 (11)	C3—O1—H1	110.2 (12)
O2—C4—C5	118.66 (11)	C4—O2—H2	107.7 (12)
O2—C4—C3	121.50 (11)	C5—O3—H3	109.9 (12)
C5—C4—C3	119.85 (11)	C7—O4—H4	110.8 (12)
O3—C5—C6	118.73 (11)		
			
C6—C1—C2—C3	0.27 (19)	O2—C4—C5—C6	−178.95 (11)
C7—C1—C2—C3	−179.80 (11)	C3—C4—C5—C6	1.06 (19)
C1—C2—C3—O1	−179.47 (12)	O3—C5—C6—C1	−178.09 (11)
C1—C2—C3—C4	1.08 (19)	C4—C5—C6—C1	0.28 (19)
O1—C3—C4—O2	−1.25 (18)	C2—C1—C6—C5	−0.96 (19)
C2—C3—C4—O2	178.26 (11)	C7—C1—C6—C5	179.12 (11)
O1—C3—C4—C5	178.74 (11)	C6—C1—C7—O5	0.77 (19)
C2—C3—C4—C5	−1.75 (19)	C2—C1—C7—O5	−179.16 (12)
O2—C4—C5—O3	−0.62 (18)	C6—C1—C7—O4	−179.37 (11)
C3—C4—C5—O3	179.39 (11)	C2—C1—C7—O4	0.70 (17)

### Hydrogen-bond geometry (Å, °)

**Table:**

*D*—H···*A*	*D*—H	H···*A*	*D*···*A*	*D*—H···*A*
O2—H2···O1	0.82 (1)	2.19 (2)	2.6625 (14)	117 (1)
O3—H3···O2	0.82 (1)	2.35 (2)	2.7464 (14)	110 (1)
O1—H1···O5^i^	0.84 (1)	1.89 (2)	2.7324 (13)	176 (2)
O3—H3···O3^ii^	0.82 (1)	2.04 (2)	2.8167 (9)	157 (2)
O4—H4···O5^iii^	0.85 (2)	1.81 (2)	2.6570 (13)	175 (2)

Symmetry codes: (i) *x*, −*y*+1, *z*+1/2; (ii) −*x*+1/2, *y*+1/2, −*z*−1/2; (iii) −*x*+1, −*y*, −*z*.
